# Acceptability and Usability of a Digital Behavioral Health Platform for Youth at Risk of Suicide: User-Centered Design Study With Patients, Practitioners, and Business Gatekeepers

**DOI:** 10.2196/65418

**Published:** 2025-05-02

**Authors:** Trinity Chloe Tse, Lauren S Weiner, Carter J Funkhouser, Danielle DeLuise, Colleen Cullen, Zachary Blumkin, Casey O'Brien, Randy P Auerbach, Nicholas B Allen

**Affiliations:** 1 Division of Child and Adolescent Psychiatry Columbia University Irving Medical Center Columbia University New York, NY United States; 2 New York State Psychiatric Institute Manhattan, NY United States; 3 Ksana Health Inc Eugene, OR United States; 4 Department of Psychology University of Oregon Eugene, OR United States

**Keywords:** user-centered design, mobile sensing, behavior change, suicide prevention, digital health, mental health

## Abstract

**Background:**

Youth suicide rates are climbing, underscoring the need to improve clinical care. Personal smartphones can provide an understanding of proximal risk factors associated with suicide and facilitate consistent contact between patients and practitioners to improve treatment engagement and effectiveness. The Vira digital behavior change platform (Vira) consists of a patient smartphone app and a web-based practitioner portal (Vira Pro) that integrates objective mobile sensing data with Health Insurance Portability and Accountability Act (HIPAA)–compliant communication tools. Through Vira, practitioners can continuously assess patients’ real-world behavior and provide clinical tools to enhance treatment via just-in-time behavior change support.

**Objective:**

This study aimed to explore the acceptability and usability of the minimal viable product version of Vira through a user-centered design (UCD) approach and to identify barriers to implementing Vira in the context of an adolescent intensive outpatient program.

**Methods:**

Over 2 iterative phases, feedback was gathered from adolescent patients (n=16), mental health practitioners (n=11), and business gatekeepers (n=5). The mixed methods UCD approach included individual semistructured interviews (eg, perspectives on treatment and attitudes toward digital tools), surveys (eg, usability), and unmoderated user testing sessions (eg, user experience).

**Results:**

Overall, participants expressed optimism regarding Vira, particularly among adolescents, who showed high satisfaction with the app’s interface and design. However, clinicians reported more mixed views, agreeing that it would be useful in treatment but also expressing concerns about the volume and displays of patient data in Vira Pro, workload management, and boundaries. Gatekeepers identified usability issues and implementation barriers related to electronic health records but also recognized Vira’s potential to enhance treatment outcomes. Feedback from stakeholders informed several crucial changes to the platform, including adjustments to data-sharing protocols, user interface enhancements, and modifications to training methods.

**Conclusions:**

Vira has a high potential to improve patient engagement and improve clinical outcomes among high-risk youth. Iterative UCD and ongoing stakeholder engagement are essential for developing technology-based interventions that effectively meet the needs of diverse end users and align with clinical workflows.

## Introduction

### Background

Suicide is the second leading cause of death among adolescents, and from 2011 to 2021, the rate of suicide attempts and suicidal behaviors has been increasing [1-3]. Existing mental health treatments are only moderately effective [4], and even among those who receive a “gold-standard” treatment, many do not improve. To reduce the public health burden of adolescent mental health problems, there is a critical need for novel approaches that increase the effectiveness of existing treatments.

One promising way to improve mental health treatments is to leverage digital devices such as personal smartphones. Smartphones have the potential to both monitor risk in real time, and to deliver just-in-time interventions, both of which are difficult if not impossible to achieve without these ubiquitous personal computing devices. To date, few digital interventions have been developed with or for youth at risk of suicide and rigorously tested to provide evidence of feasibility, acceptability, or efficacy [5,6]. Examples of other mental health apps targeted toward mitigating suicide risk include BRITE, LifeBuoy, and BlueIce. BRITE was developed to support suicide safety planning among adolescents consists of a practitioner portal and patient app, and has primarily been implemented as a single-session intervention [7]. By contrast, LifeBuoy is an app that helps young people manage suicidal thoughts in their daily lives by teaching dialectical behavior therapy (DBT) skills via self-guided modules [8], whereas BlueIce (MyOxygen, Limited) is used to support distress tolerance and reduce self-harm through a personalized toolbox of strategies that include mood diaries, a menu of mood-lifting activities, and automatic routing to emergency numbers if needed [9]. Although initial clinical outcomes are promising [10], these apps do not appear to support continuous treatment between patients and their practitioners, a critical aspect of evidence-based treatments such as DBT [11], nor do they include mobile sensing, which could provide unique insights into risk processes and opportunities for intervention that occur outside the health care context.

Measurement-based care is important for monitoring treatment effectiveness and improving clinical outcomes [12] but is not often used in routine clinical care due to concerns about practitioner and patient burden [13]. Through both passive (eg, built-in smartphone sensors) and active data collection (eg, experience sampling methods), smartphones can directly address these concerns by allowing practitioners and patients to monitor mood, behavior, symptoms, and risk factors continuously, objectively, and with a relatively low burden. In addition, smartphones afford novel opportunities to reinforce or introduce intervention content between sessions. For example, patients often forget to practice therapeutic skills outside of sessions [14], which may lead to poorer treatment outcomes [15]. Smartphone push notifications delivered between sessions can remind patients to practice therapeutic skills, thus increasing skill usage and improving patient outcomes [16].

Although digital mental health interventions have significant potential, low adherence and high attrition can reduce clinical effectiveness [17]. In particular, digital interventions that do not address the unique needs and perspectives of end users may exhibit low usability, which leads to lower sustained engagement [18]. When developing digital interventions, it is important to consider the clinical and implementation context [19,20]. User-centered design (UCD) uses an iterative, stakeholder-engaged process to cocreate products that directly respond to the end user experience [21,22]. Through UCD, individuals with lived experiences are closely engaged during the design and development processes by providing feedback that can be used by intervention developers and designers to tailor the content, interface, and implementation of digital interventions [23,24]. UCD studies also inform the translation of evidence-based treatments to digital formats and reveal critical information about facilitators and barriers to adoption that guide implementation approaches [25,26].

**Figure 1:**
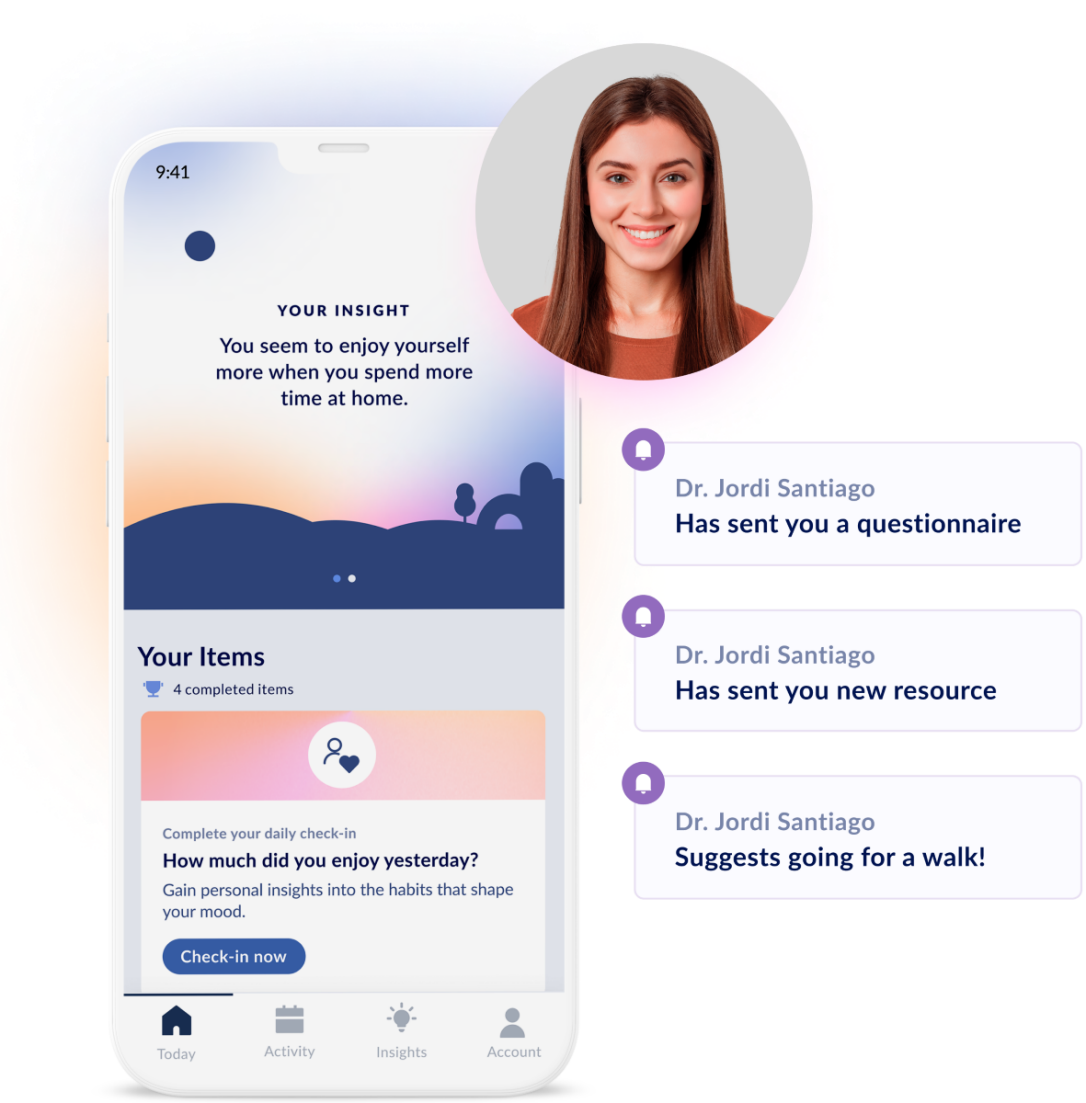
The Vira digital behavior change platform consists of a user-facing smartphone app, Vira, and a linked, web-based practitioner portal, Vira Pro.

The Vira digital behavior change platform consists of a user-facing smartphone app and a web-based linked practitioner portal, Vira Pro (refer to Figure 1). The Vira platform was developed based on principles of behavioral activation, an effective treatment for depression and other mental health problems [27]. The smartphone app uses mobile sensing and brief self-report surveys to monitor numerous indicators of mental health problems (eg, sleep disturbance and low mood) or proximal risk factors. The app provides patients access to these data 24/7 and offers insights into passively sensed behavioral patterns associated with their moods. The web portal displays these data to the clinician, allowing them to gain more nuanced insights into their patients’ behavior and mood between sessions. Clinicians can also use the web portal to schedule personalized push notifications to be delivered to a patients’ smartphone, thereby facilitating enhanced support between sessions. Importantly, this functionality allows pushing notifications to be delivered at the “right time” (eg, as just-in-time adaptive interventions), which may improve outcomes [28].

### Study Objectives

This study aimed to evaluate the Vira mobile app and Vira Pro practitioner portal for use with youth at high risk for suicide by using an iterative formative development process to incorporate feedback from adolescents with lived experience, care providers, and business gatekeepers (ie, individuals who are responsible for making decisions regarding financial and other support for clinic initiatives). By including the perspectives of these end users, we sought to develop a digital platform that can be integrated into clinical workflows, is maximally engaging, and optimized for successful implementation.

## Methods

### Study Overview

**Figure 2:**
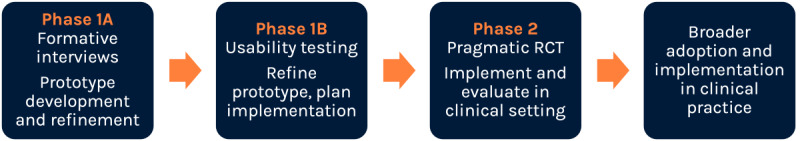
Overview of the iterative formative development process and overall project design. RCT: randomized controlled trial.

This study was part of a larger project to develop and test a behavior change platform for use in an intensive outpatient program (IOP) serving youth at high risk of suicide. The goal of the iterative formative development study, which consisted of 2 phases of mixed methods data collection, was to inform product development and implementation for a pragmatic randomized controlled trial (RCT; refer to Figure 2 for study design) [29].

### Participant Recruitment

Participants were recruited between October 2022 and March 2023. Three types of stakeholders were recruited from the broader NewYork-Presbyterian Hospital, ColumbiaDoctors, and New York State Psychiatric Institute communities to participate across 2 iterative phases of research (Phase 1 and Phase 2). Stakeholders included adolescents who had received previous mental health treatment, mental health clinicians, and business gatekeepers.

Adolescent participants were recruited from a pool who had previously participated in other studies and provided informed consent to be contacted for future study opportunities. Adolescents were considered eligible if they were currently receiving or had received mental health treatment for depression or anxiety symptoms within the past year, were between the ages of 13 and 18 years old, and were fluent in English.

Clinicians and business gatekeepers were recruited internally via outreach emails and electronic flyers. Eligibility criteria for clinicians and business gatekeepers included present employment in youth mental health services at NewYork-Presbyterian Hospital, ColumbiaDoctors, or New York State Psychiatric Institute, ages 18-56 years old, and fluency in English. Business gatekeepers served in clinical leadership roles (eg, clinic director and executive director). Several clinicians who participated in Phase 1 also participated in Phase 2. One participant held both administrative and clinical roles and therefore participated as a business gatekeeper in Phase 1 and a clinician in Phase 2. Several of the clinicians and business gatekeepers have previously collaborated with RPA on other research projects.

### Study Procedures

This study was an iterative formative development process consisting of 2 phases of mixed methods data collection. A UCD approach was adopted whereby changes made to the app prototype presented in Phase 2 were based on participant feedback from Phase 1. In both phases, participants completed semistructured interviews, an unmoderated click-through and think-aloud exercise to provide feedback on the minimal viable product version of the app, and a quantitative feedback survey using items adapted from the technology acceptance model (TAM) [30]. The TAM has 2 scales to measure specific beliefs that may influence the acceptance of technology: perceived usefulness and perceived ease of use. Each scale contains 10 items, which are rated using a 7-point Likert scale from 1 (strongly disagree) to 7 (strongly agree). Higher ratings indicated higher acceptability. Participants in both phases also completed a demographics questionnaire. Each interview was conducted by 2 moderators (TCT and DD). Both moderators are female and hold bachelor’s degrees. TCT is a research assistant, and DD oversaw product design and development at Ksana Health, Inc at the time of the study.

### Phase 1

Formative interviews in Phase 1 aimed to increase our understanding of the target patient population and clinical context, elicit feedback on the Vira minimal viable product to inform adaptations and refinements to the platform to increase relevance for the IOP clinical setting, promote perceived ease of use, understand potential barriers, and facilitate future implementation. Adolescents, clinicians, and gatekeepers were included in this phase.

Adolescent interview topics in Phase 1 included their past and current experiences receiving mental health treatment, smartphone usage and habits, and initial impressions of Vira based on a verbal description of the platform. Clinician interviews focused on their current therapy practices, experience using technology as part of the mental health treatment they deliver, past experiences integrating digital tools into existing electronic health record (EHR) systems, and their first impressions of Vira. Business gatekeepers reported on their experiences managing clinicians and clinic caseload, use of technology as part of mental health treatment, past experiences integrating new tools into existing EHR systems, and their first impressions of the platform. Interviews were approximately 30 minutes in duration and were conducted through Microsoft Teams videoconferencing software.

After completing the interview, participants took part in an unmoderated user testing session. Usability testing was conducted through UserTesting and began with a “think-aloud” procedure to test user interactions with Vira [31,32]. Participants were asked to verbalize their reactions as they viewed screens and to describe their navigational choices as they used the platform. Users were also asked about barriers and facilitators to using the platform, perceptions of specific features, and how to improve it.

### Phase 2

The goal of Phase 2 usability testing was to understand the usability and acceptability of the refined Vira platform and deepen our knowledge of the implementation context to support clinical implementation in the subsequent pragmatic RCT. Adolescents and clinicians were included in this phase, as they were the primary target end users in the subsequent pragmatic RCT.

Phase 2 interviews with adolescents and clinicians explored similar topics to the Phase 1 interviews. In Phase 2, clinician interviews additionally focused on implementation, clinician readiness, and usability of the revised Vira platform. Interviews necessitated approximately 30 minutes and were completed through Microsoft Teams and Zoom. During the unmoderated user testing portion of the session completed through the UserTesting platform, clinicians were provided with a mock patient profile and asked to think aloud as they explored the Vira tool as if they were using it in a client session. The goal of this mock case study testing format was to understand the clinical implementation context, facilitators and barriers to use, and overall usability. Clinicians were also asked more detailed questions about EHR integration, including which data points from Vira would be most useful to them.

### Ethical Considerations

This study was reviewed and approved by the New York State Psychiatric Institute Institutional Review Board (protocol #8307). Signed electronic informed consent was obtained from all participants included in this study. The study data were anonymized and deidentified. All data is uploaded to a secure research server that is only accessible by the research team. Participants were compensated US $200 for their participation in the study.

### Data Analysis

Similar analytic approaches were used across phases. Qualitative and quantitative data were analyzed separately.

#### Semistructured Interviews and UserTesting Transcripts

Semistructured qualitative interviews and UserTesting transcripts were analyzed using a thematic analysis approach [33]. Coding categories were developed to characterize participant responses, including reactions to Vira’s features and functions as well as overall difficulty or facility using the platform. Given the iterative nature of the project, rapid analysis techniques, including matrix displays and triangulation [34,35], were also used. Multiple team members (TCT, LSW, DD, RPA, and NBA) reviewed transcripts to identify themes across phases for each persona (adolescent, clinician, or gatekeeper). Once team consensus was reached, findings were summarized narratively by theme and persona.

#### Quantitative Surveys

Demographics were analyzed using descriptive statistics and frequencies (mean [SD] and n [%] where applicable). TAM ratings were analyzed by comparing frequencies (mean [SD]) and n (%) of ratings within and across phases.

#### Mixed Methods Interpretation

The 2 sets of results (qualitative and quantitative) for each persona were triangulated by theme across both phases to examine how each set of data compared and contrasted to the other set and to explore the complementarity of the results across data collection methods. The qualitative and quantitative results were then synthesized for interpretation. The mixed methods synthesis of qualitative and quantitative results across both phases is presented by persona in the *Results* section.

## Results

### Demographics

A total of 36 participants were recruited across both phases of the study. In Phase 1, a total of 8 adolescents, 7 clinicians, and 5 gatekeepers participated in stakeholder consultations. One gatekeeper did not provide demographic information. In total, 4 of the clinicians in Phase 1 also participated in Phase 2. One gatekeeper participated in Phase 2 as a clinician. A total of 8 adolescents and 7 clinicians participated in Phase 2. Refer to Table 1 for sample characteristics.

Table 2 summarizes participant responses to the TAM. One gatekeeper did not complete TAM in Phase 1 and one clinician did not complete TAM in Phase 2. For individual item frequency distributions, please refer to Figures S1-S6 in the Multimedia Appendix 1.

**Table 1:** Demographic characteristics of participants (N=36).

				Phase 1		Phase 2
Adolescents, n				8		8
Age (years), mean (SD)				16.2 (0.91)		17.5 (0.91)
Women, n (%)				7 (87.50)		7 (87.50)
**Race, n (%)**						
	White			3 (37.50)		5 (62.50)
	Asian			1 (12.50)		2 (25.00)
	Black			0 (0)		1 (12.50)
	More than one race			3 (37.50)		0 (0)
	Unknown or not reported			1 (12.50)		0 (0)
**Ethnicity, n (%)**						
	Hispanic			1 (12.50)		0 (0)
	Non-Hispanic			7 (87.50)		8 (100)
**Clinicians (N)**				7		8
	Age in years, mean (SD)			32.14 (5.24)		34.75 (4.92)
	Women, n (%)			7 (100)		8 (100)
**Race, n (%)**						
	White			5 (71.43)		7 (87.50)
	More than one race			2 (28.57)		1 (12.50)
	**Ethnicity, n (%)**					
		Hispanic	1 (14.29)		0 (0)	
		Non-Hispanic	6 (85.71)		8 (100)	
**Business gatekeepers (N)**				5		—^a^
	Age (years), mean (SD)			44.6 (9.09)		—
	Women, n (%)			2 (40)		—
**Race, n (%)**						
	White			4 (80)		—
	Unknown or not reported			1 (20)		—
**Ethnicity, n (%)**						
	Non-Hispanic			4 (80)		—
	Unknown or not reported			1 (20)		—

^a^Not applicable.

**Table 2:** Participant technology acceptance model responses (N=33).

Technology acceptance model			Adolescents, mean (SD)				Clinicians, mean (SD)				Gatekeepers, mean (SD)
			Phase 1 (n=8)		Phase 2 (n=7)		Phase 1 (n=7)		Phase 2 (n=7)		Phase 1 (n=4)
**Perceived usefulness scale**											
	Greater control	5.63 (0.74)		5.14 (1.35)		4.86 (0.69)		5.14 (1.68)		6.00 (0.82)	
	Improve quality	6.13 (0.64)		5.14 (1.86)		4.86 (0.90)		5.00 (2.16)		6.50 (0.58)	
	Accomplish quickly	5.50 (1.31)		5.86 (1.21)		3.86 (2.12)		4.71 (1.38)		5.75 (1.50)	
	Support critical aspects	5.75 (1.28)		5.29 (1.25)		5.00 (0.82)		5.43 (1.62)		6.50 (0.58)	
	Productive	5.88 (0.99)		5.71 (1.38)		3.86 (1.07)		4.43 (2.30)		5.25 (2.06)	
	Improve	5.75 (1.28)		5.57 (1.40)		4.43 (1.62)		5.14 (1.68)		5.75 (0.96)	
	Accomplish more	6.00 (1.20)		5.43 (1.40)		5.14 (1.21)		3.29 (2.14)		5.50 (1.29)	
	Enhance effectiveness	6.00 (1.31)		5.57 (1.40)		5.00 (0.82)		5.14 (1.95)		6.50 (0.58)	
	Easier	5.75 (1.49)		4.71 (1.25)		3.86 (1.77)		4.57 (1.40)		5.50 (1.29)	
	Useful	6.00 (0.93)		5.43 (1.13)		5.00 (1.00)		5.86 (1.21)		6.25 (0.50)	
**Perceived ease of use scale**											
	Cumbersome to use	2.71 (1.38)		3.14 (1.86)		3.43 (1.27)		4.71 (2.14)		2.00 (1.41)	
	Easy to learn	5.75 (1.48)		5.57 (0.98)		5.71 (1.11)		4.86 (0.90)		6.00 (0.82)	
	Frustrating	2.63 (1.60)		2.29 (1.11)		3.00 (1.15)		2.86 (1.07)		2.00 (0.82)	
	Confusing	2.50 (1.41)		2.43 (1.51)		3.29 (1.60)		2.71 (1.38)		2.00 (0.82)	
	Rigid and inflexible	2.00 (1.31)		2.29 (1.60)		3.14 (1.68)		2.00 (1.00)		2.75 (1.50)	
	Easy to remember	5.38 (1.92)		5.29 (1.11)		5.57 (0.53)		4.71 (1.80)		6.50 (0.58)	
	A lot of mental effort	3.38 (1.19)		4.00 (2.24)		3.86 (1.35)		3.57 (1.51)		2.25 (1.50)	
	Easy to understand	6.13 (1.13)		6.00 (1.00)		5.57 (0.53)		4.86 (0.69)		5.00 (2.45)	
	Easy to become skillful	6.38 (0.52)		5.29 (1.70)		5.29 (0.76)		5.00 (1.00)		6.25 (0.96)	
	Easy to use	6.25 (0.71)		5.86 (1.21)		5.57 (0.53)		5.43 (1.13)		6.00 (1.15)	

### Adolescents

#### Passive Sensing Data

The overall concept of using mobile sensors to passively capture data about their behavior was intriguing to all participants. Adolescents felt that the passive sensing data would be more accurate than their own self-reports in reflecting their sleep and fitness habits and that this technology could improve the efficiency of their therapy sessions by allowing them to easily share behavioral data with their clinician. Adolescents also thought that Vira would improve the quality of their treatment and support critical aspects of it (TAM perceived usefulness items 2 and 4; refer to Table 2).

Adolescents across both phases had mixed reactions to the different types of data that would be collected by the Vira app via mobile sensors. Most adolescents were excited for the app to track their sleep, walking, and exercise patterns, and identified these behaviors as factors that impact mental health but that they are often not aware of or tracking on their own. They were also enthusiastic about their clinicians accessing these data through Vira and using it to support their treatment. Refer to exemplar testimonials in Textbox 1.

However, participants were more apprehensive about sharing location (GPS) data. Many were confused about how location data sharing would work and expressed that they did not want their therapist to actively track their live location (even though this is not a capability of the app). One adolescent expressed that their data-sharing preferences could vary based on their emotional state. There were also some concerns regarding how language patterns derived from keyboard data would be interpreted by clinicians.

I feel like people’s words and conversations can be interpreted in many ways, and I don’t feel like statistically, they’ll be an accurate representation.

ID A13, Phase 2

**Passive sensing data feedback.**

- *Having a record of things like sleep and physical activity can help you because these are things that sometimes we do not think twice about. But they have a huge impact on how we are feeling so to have a record and to also be able to have that type of platform to communicate with your therapist sounds really efficient.* [ID A08, Phase 1]
- *My therapist seeing that I am not sleeping well would be helpful, it will save time in therapy and allow for deeper and more helpful conversations rather than recapping.* [ID A03, Phase 1]
- *If it is about sleep and your physical activity, that is something that can affect your mental health. So, it is not like it is taking unnecessary information. It is just like more information that you may not even think to bring up in your session because it is just part of your daily life.* [ID A15, Phase 2]

#### Home Page Feedback

Adolescents responded positively to the app’s home page interface, stating it was easy to navigate and aesthetically pleasing. Most adolescents perceived it as easy to learn and understand (TAM perceived ease of use items 2 and 8). Most adolescents also liked the sample psychoeducation articles that were displayed on the home page.

However, some adolescents expressed concern about engagement with the app given that personalized insights about connections between their mood and their passively sensed behaviors would not be available until they reported their daily mood for 10 days. Adolescents suggested that having more features available during the period before insights are available would increase app engagement.

#### Communicating With Clinicians Between Sessions and Nudges

Adolescents liked the idea of clinicians using Vira to send nudges to them between sessions, particularly psychoeducational videos, reminders to use coping skills or general mood boosters.

It would be nice to have a therapist who knew how I felt even on the days that we didn’t meet and could send me a little something.

ID A02, Phase 1

However, there were a few adolescents who expressed concern about the potential intrusiveness of between-session contact. They were also concerned with the nuisance of receiving too many smartphone notifications.

Several adolescents in Phase 2 felt that the frequency of between-session communication should be variable based on a few factors, including the strength of the relationship and their current symptom severity. Adolescents expressed that they would feel more comfortable engaging in more frequent communication with their therapist between sessions only once they had built a good rapport and relationship with them.

### Clinicians

#### Using Vira in the Clinical Workflow

Clinicians consistently expressed that they are seeking a tool that facilitates their workflow and makes their job easier, not harder. Clinicians explained that they have many responsibilities and take on multiple roles (eg, direct service provider and clinic administrator), and thus need a tool that will lend itself to more efficient preparation before client sessions.

Clinicians interviewed in Phase 1 felt the Vira Pro interface was overwhelming, with potentially too much data provided. One clinician felt that using Vira could slow down their workflow.

It would make me accomplish tasks more slowly because there’s so much more data.

ID C02, Phase 1

Between the Phase 1 and Phase 2 tests, changes were made to the prototypes to simplify the data display in Vira Pro. Yet, clinicians interviewed in Phase 2 also felt the large volume of data was challenging, and like clinicians interviewed in Phase 1, felt that trying to extract clinically relevant components and apply them to their sessions could create extra work.

I guess I have a little bit of a worry of how do I incorporate all this data in a way that doesn’t feel like a waste of time for the patient but isn’t creating a lot of extra work for me compared to what I’m doing today?

ID C11, Phase 2

Some clinicians suggested that more concise data summarization within Vira Pro would make the tool more usable. Despite their comments about the interface being overwhelming, clinicians across both phases felt that Vira would be easy to learn and use (TAM perceived ease of use items 2 and 10).

Although all clinicians across both phases felt that Vira would be useful to their clients’ treatment (TAM perceived usefulness item 10), there were mixed opinions on whether Vira would increase their efficiency and job performance. The majority of clinicians felt that Vira would support critical aspects of their job and enhance their effectiveness on the job, but there were differences in perceptions of whether using Vira would allow them to accomplish more tasks quickly and increase their job productivity (TAM perceived usefulness items 3 and 5). Interestingly, fewer of the Phase 2 clinicians felt that Vira would allow them to accomplish more in their job than would otherwise be possible compared to Phase 1 clinicians (TAM perceived usefulness item 7).

#### Nudges

Clinicians had mixed opinions on the utility of scheduling nudges to practice therapy techniques. Some reacted very positively to the feature and felt that it could be useful to their patient’s treatment by reinforcing the use of therapy principles discussed in the session in the context of the patient’s everyday life.

I love the idea of the nudges, and I’m thinking how instrumental that would be in transforming phone coaching and generalizing any sort of DBT skills to the environment.

ID C03, Phase 2

However, other clinicians felt that nudges between sessions may lead patients to become too reliant on their therapist and neglect to engage in therapeutic strategies on their own, a key step in sustained behavior change. One clinician felt that it was unnecessary to use nudges to remind patients to use therapeutic techniques, preferring instead to simply speak to the patient in person about using them.

And then nudging, I’m not particularly fond of, because I feel like it could potentially reinforce passivity for a patient. If a provider is nudging them and reminding them to do things, it’s kind of taking away the autonomy that they can do it on their own.

ID C04, Phase 2

I’m just imagining it’s us sitting there monitoring our patients and nudging them to use skills [...] and it fragilizes them and doesn’t hold them accountable for being responsible for their own well-being.

ID, C02, Phase 2

Within the Vira Pro dashboard, all clinicians agreed that the section showing “default” nudges should be adapted for each client’s clinical needs. For example, the most common therapeutic modalities and techniques used for a patient with anxiety are different from those used for a patient with depression.

I would want to be able to customize it for the client because I think it would make a difference based on what I was working with the client on. [...] So they might vary depending on the focus of the treatment.

ID C09, Phase 2

#### EHR Integration

Clinicians offered several suggestions related to integrating the Vira platform into EHR systems. They frequently stated that having the data that is collected in Vira (eg, passive sensing data and assessment responses) automatically populate into their EHR would reduce the burden of manually uploading data. Similarly, clinicians thought that it would be efficient to have clinical notes generated in Vira appear in their EHR system, reducing the need for them to rewrite their notes in multiple locations.

Minimal effort on my end to change anything. Minimal training. Nothing that I have to do too much work on my end to upload [Vira data].

ID C04, Phase 1

To support seamless integration, several clinicians felt it would be important to reduce the number of redundant logins needed to access Vira within their EHR. Clinicians interviewed in Phase 2 considered the following types of Vira data most important for EHR integration: daily self-reported mood check-ins, assessment responses and scores, and the Vira-derived associations between self-reported mood and client behavior.

### Business Gatekeepers

#### Overview

All gatekeepers felt that Vira would be a useful tool for their team’s treatment toolkit (TAM perceived usefulness item 10). All gatekeepers felt that using Vira would improve the quality of the work they do (TAM perceived usefulness item 2), support critical aspects of their job (TAM perceived usefulness item 4), and enhance their effectiveness on the job (TAM perceived usefulness item 8). They also agreed that Vira seems easy to learn (TAM perceived ease of use item 2), easy to become skillful at using (TAM perceived ease of use item 9), and easy to use (TAM perceived ease of use item 10).

#### Vira Pro Interface

Through the user testing sessions, administrators provided feedback about the usability and acceptability of several aspects of the Vira Pro administrator dashboard. Gatekeepers responded positively to the visual layout and liked that they could access information about all patients in their clinic from a single page within the administrator dashboard. They also expressed that it would be important for the results of patient’s risk assessments to be displayed within the dashboard alongside the other assessments that have been completed so that they are easily accessible for practitioners.

There were several aspects of the practitioner platform that gatekeepers were unclear about. Within the administrator dashboard, it was unclear to them where the data for “Top 10 Used Intervention Techniques” is derived from and that it is a compilation of all the clients within the clinic. Gatekeepers also sought more detailed explanations of the Vira metrics within the dashboard, such as how language categories are defined and examples.

I would love to see how these categories of language are defined in really clear ways. Like ‘rigid words’ for example, I’d love to see some examples.

ID G2, Phase 1

#### Implementation Considerations

During the interviews, gatekeepers were asked to consider several aspects of implementing Vira within their clinic. First, gatekeepers felt that Vira would be helpful because it could inform clinic treatment planning.

Gatekeepers were asked which clinicians would find Vira most helpful. All participants stated that Vira would be a useful tool for clinicians who employ cognitive behavioral therapy and DBT within their treatment framework, as Vira’s functionalities could enhance their ability to practice those treatment modalities.

Folks who are involved in DBT and CBT could really be the ones that could utilize the tool because it most mimics what we already do, right? CBT, it’s all about the exposures so how could we use Vira to help with the exposures. With DBT, it’s all about practicing those skills and some of the skills are about sleeping well, taking your medication, exercising or just doing any sort of physical activity, all those things. So again, I think in some ways, this would really complement those types of providers.

ID G2, Phase 1

Gatekeepers felt Vira might be useful for patients experiencing depression and/or anxiety. One gatekeeper mentioned Vira potentially being useful for patients with physical health comorbidities due to Vira’s health behavior metrics and expressed interest in integrating Vira with primary care or other medical departments.

Gatekeepers also brought up several potential barriers to implementation. The most common concern was the confidentiality of patient data collected from Vira. There were also some concerns about the extent of parental involvement, including how much Vira data they could access if patients are minors. Some gatekeepers were also worried that implementing Vira could be potentially burdensome for providers by adding to their workload, depending on clinic expectations.

I do worry that it would be burdensome to the provider if there’s any expectations around how much they’re supposed to be babysitting, so to speak.

ID G3, Phase 1

Another implementation roadblock gatekeepers identified is getting clinicians to try a new tool and do something different in their treatment. One gatekeeper suggested incorporating multimodal training to help clinicians transition to using the tool could help overcome this obstacle. Gatekeepers also foresaw difficulties in integrating Vira with existing EHR systems.

## Discussion

### Principal Findings

This study used UCD methods to garner feedback about the Vira behavior change platform from 3 groups of key stakeholders: adolescent patients, clinicians, and business gatekeepers. All participants expressed that Vira could be useful in enhancing mental health treatment. Adolescents had positive feedback on the aesthetics and design of the Vira app, factors that may promote engagement with mental health apps for youth at risk of suicide [36]. Overall, clinicians felt that the displays in the Vira Pro practitioner portal were too dense due to the volume of data shown and had mixed feedback about how using Vira would impact their efficiency and productivity. Clinicians were generally open to the idea of using Vira to send nudges to clients between sessions but expressed some concerns. Gatekeepers had mixed feedback on the usability of the tool but felt that Vira would enhance their effectiveness on the job and shared valuable feedback to inform implementation.

### General Capabilities and Data Sharing

All participants expressed excitement about Vira’s passive sensing capabilities to track behavior and the daily mood. This aligns with UCD research conducted as part of the development of the BlueIce app, which similarly found that adolescents were enthusiastic about tracking mood patterns and noticing their patterns, as well as identifying triggers of negative moods, similar to Vira’s insights [37]. However, there was some apprehension about potential intrusiveness to patients. Specifically, adolescents were most worried about sharing location data and not wanting to be actively tracked by their therapist, and clinicians were only interested in accessing information that adolescents were enthusiastically willing to share with them. To address adolescents’ concerns about collecting and sharing location and keyboard data, it will be important to provide them with detailed information and instructions on how their data is processed, stored, and used by the app and their clinician at the time of installation. Specifically, clients should be informed that clinicians do not have real-time access to behavioral data, nor do they have specific details about exact locations or words typed into their smartphones. Taken together, these findings suggest that clear alignment between patients and providers on which data they are comfortable sharing, and what exactly is shared, is important for aiding clinicians and clients in leveraging passive sensing data in therapy and maintaining supportive therapeutic relationships.

### Communication Between Sessions (Nudges)

The Vira tool allows clinicians to send prescheduled notifications to patients to support compliance with their therapy plans. By collecting passive sensing data that is displayed in both the Vira app and on the practitioner dashboard, Vira also provides information about client behavior continuously between sessions. In this study, adolescents and clinicians expressed mixed views on whether these features would improve treatment. While some adolescents were enthusiastic, others noted that it might be frustrating to receive too many notifications from their therapist. This aligns with feedback from target adolescent end users of the LifeBuoy app regarding the frequency of app notifications [36]. Several clinicians in this study shared similar concerns.

Stakeholder feedback from this study therefore suggests it is essential to set clear expectations with both adolescents and practitioners about how that between-session communication will be operationalized. Gatekeepers felt the tool would be a supportive addition to their team’s treatment toolkit, but were concerned about the potential burden on clinicians, underscoring the importance of clearly defining expectations with providers. Along similar lines, when introducing the tool to their patients, practitioners should discuss both the frequency with which they will use Vira and how they will use it to support treatment. Similar to Hetrick et al [38], clinicians in our study expressed that although the tool could be useful in monitoring patients’ moods, monitoring patient data multiple times between sessions or in real time is not feasible. However, to be clear, this is not an expectation of typical use of the Vira Platform. Ensuring the tool is designed to fit within clinician workflows and managing patients’ expectations for how their clinician will use it increases the likelihood of clinician adoption [39].

Adolescents were very enthusiastic about Vira’s nudges. They uniformly liked the concept of clinicians using the passive sensing data to determine what types of nudges should be sent to enhance their treatment. They also felt it would be helpful for clinicians to send nudges as reminders to use therapeutic techniques learned during treatment. Conversely, clinicians had mixed opinions on the nudging feature. Some clinicians felt nudges could be very useful and could transform treatment in a positive way, consistent with recent research demonstrating that nudges and prompts increase engagement in self-guided interventions [40]. However, it is important to note that patients in the IOP setting would be using Vira to support DBT, a collaborative and therapist-guided treatment modality. Other clinicians expressed concern that patients may become dependent on their clinician to send nudges to remember to use therapeutic techniques discussed in sessions. In sum, Vira’s nudge capability may help promote habit formation in terms of therapy techniques and coping skills outside of the therapy sessions, and it is important that digital products allow clinicians to schedule and structure nudges in line with each patient’s personalized and dynamic treatment needs. For example, clinicians may schedule nudges to be delivered less frequently as skills are acquired and used.

### Product Changes

Findings from this iterative UCD process directly informed the development of several changes to the Vira platform that will be implemented for the pragmatic RCT. In response to feedback about data sharing, app users will have the flexibility to choose which data streams they would like to share with their practitioner, including specific streams of passively sensed behavioral data and daily mood (enjoyment) ratings. Enhancements have also been made to the app to boost engagement during the initial 10-day period in which Vira collects sufficient information to calculate personalized insights about behaviors that may be related to a user’s mood. In the version of the app that was tested in this study, personalized insights were not available to users until their 10th daily check-in. Based on insights from this UCD study, the app has been updated such that users receive more onboarding information within the app after their third daily check-in, and insights about the consistency of their sleep and walking patterns after their sixth and eighth check-ins, respectively.

Clinicians and gatekeepers expressed strong desires for deeper information about the various sensor streams and data types available within the dashboard, as well as a simpler and more accessible user interface. Therefore, the Vira Pro dashboard was modified to show a more summarized, simplified form of patient data with more options to hide granularity in data charts. The revised version of the platform also retains the capability to drill into the details as needed; Vira Pro users can now hover over fields within the dashboard, such as sensor streams and specific data points, to view more details about how the data were collected and processed. Simplifying the user interface is a particularly important product change, considering that providing an overload of information can discourage clinicians from using the digital tool [41].

### Implementation Considerations

Previous research has shown that practitioners’ negative attitudes toward digital mental health interventions and resistance to change are common barriers to implementing new digital technologies in mental health care [39]. Similarly, through clinician and gatekeeper interviews, this study found that clinician buy-in with using a new tool is vital. Consistent with other research, clinicians and gatekeepers expressed concerns about the potential for mobile technology to increase professional workload [42]. Providing clinicians with sufficient training to ensure their confidence and efficiency in using the tool may increase the likelihood that clinicians integrate the tools into their practice [43], and help them overcome any concerns or hesitations they may have regarding implementation. Thorough training was also commonly suggested by gatekeepers in this study as a vital step to implementing Vira in their clinic. Further, when implementing Vira within a clinical setting, clinic administration needs to set clear expectations for how they expect Vira to be used within treatment. Moreover, clinicians and gatekeepers expressed that friction integrating Vira with existing EHR systems would be a barrier to implementation, consistent with previous research [44].

Findings from this study directly informed the development of a focused training approach that is being rolled out in the ongoing pragmatic RCT [29]. In response to clinician feedback about having limited time to learn new tools, the clinician training uses a blended synchronous and asynchronous format to provide flexibility while also keeping the training focused and efficient (1 hour total). To address mixed views regarding whether Vira would help them increase productivity, the training emphasizes critical thinking on how to apply the Vira platform to their practice. The training sequence focuses on building self-efficacy for using the platform to enhance their practice and help augment their ability to complete existing tasks. Many video and written resources are also available through a self-service knowledge base for asynchronous review and support after the training.

### Generalizability and Broader Implications

Findings from this study can be generalized to inform the development and implementation of other digital technologies for high-risk youth. Both adolescents and clinicians were enthusiastic about the potential utility of passive sensing data for treatments [37], but concerns regarding data sharing underscore the need for transparency about how data from digital health platforms are used [45].

This study also revealed important issues to consider more broadly when implementing new digital technologies into health care systems, such as the impact on clinician workloads and integrating the tool within existing clinical workflows and EHRs [39,44]. User interface design is a key consideration in increasing the acceptability of new digital technologies. Data displays should be simple and accessible so as to not overwhelm users and increase the likelihood of adoption [41].

### Limitations

This study has several limitations. Sample characteristics and recruitment methods may have impacted the feedback elicited in this study and the generalizability of the results. Many adolescents in the study had engaged in higher levels of care (eg, inpatient admission, partial hospitalization programs, and IOPs). However, the inclusion criteria for adolescent participants only required them to be currently in treatment or have received treatment in the past year for depressive or anxiety symptoms. Accordingly, some of the participants recruited may have only received a minimal level of care (ie, just a few sessions) and may have been at lower risk compared to the intended user population for Vira. In addition, the sample was limited in terms of racial and ethnic diversity, and the majority of the adolescent sample and the entire clinician sample were female. This may be because females are more likely to seek treatment for mental health [46], and the mental health workforce is predominately female [47]. Moreover, there was also a relatively small sample size. For grounded theory approaches, a sample size of 20-30 participants is generally recommended [48], although a recent systematic review found that among studies with a relatively homogeneous study population and specific objectives, a total of 9-17 interviews may be enough to reach saturation [49]. This study reached saturation with a similar sample size. Finally, we did not include parents as a stakeholder in this study. It may be useful to include parents in future studies to understand how their involvement can facilitate the implementation of digital technologies in adolescent mental health treatment.

### Future Directions

Clinician feedback about the lack of time to prepare for sessions suggests that automation may increase the acceptability and perceived usefulness of the platform. Feedback has informed the ongoing design and development of AI-supported features such as automatically generated drafts of clinical progress notes, “heads up” summaries of the most salient clinician issues that can be quickly accessed before each session, and a clinical assistant that can leverage clinical practice guidelines across a range of behavioral health conditions to suggest enhancements to therapy plans based on data collected in Vira. Moreover, the clinical effectiveness of the Vira platform is currently being investigated in a 2-arm, pragmatic RCT in which youth at risk for suicide within an IOP will be randomly assigned to use Vira with their clinician or engage in usual care [29]. Throughout this clinical trial, we will collect feedback from both patient and clinician participants.

### Conclusions

In sum, participants felt that the Vira platform has a high potential to improve engagement with mental health treatment as well as mental health outcomes in high-risk youth. Our rigorous, multimethod approach uncovered key facilitators and barriers to implementation, many of which are consistent with previous research, highlighting the challenge of translating novel, technology-based interventions from controlled to clinical settings. Applying iterative, UCD methods allowed us to integrate valuable stakeholder feedback to develop an intervention that is clinically relevant to end users, hopefully leading to successful implementation in real-world settings.

## Appendix

Multimedia Appendix 1 Additional figures.

## Abbreviations

DBT: dialectical behavior therapy
EHR: electronic health record
HIPAA: Health Insurance Portability and Accountability Act
IOP: intensive outpatient program
RCT: randomized controlled trial
TAM: technology acceptance model
UCD: user-centered design

## Data Availability

The datasets generated or analyzed during this study are available from the corresponding author on reasonable request.
