# Supplementary material for: Acceptability and Usability of a Digital Behavioral Health Platform for Youth at Risk of Suicide: User-Centered Design Study With Patients, Practitioners, and Business Gatekeepers
Source: JMIR Form Res. 2025 May 2;9:e65418. doi: 10.2196/65418 (PMC12084771; doi:10.2196/65418)

**Figure S1**. Distribution of adolescent ratings of perceived usefulness across study phases.


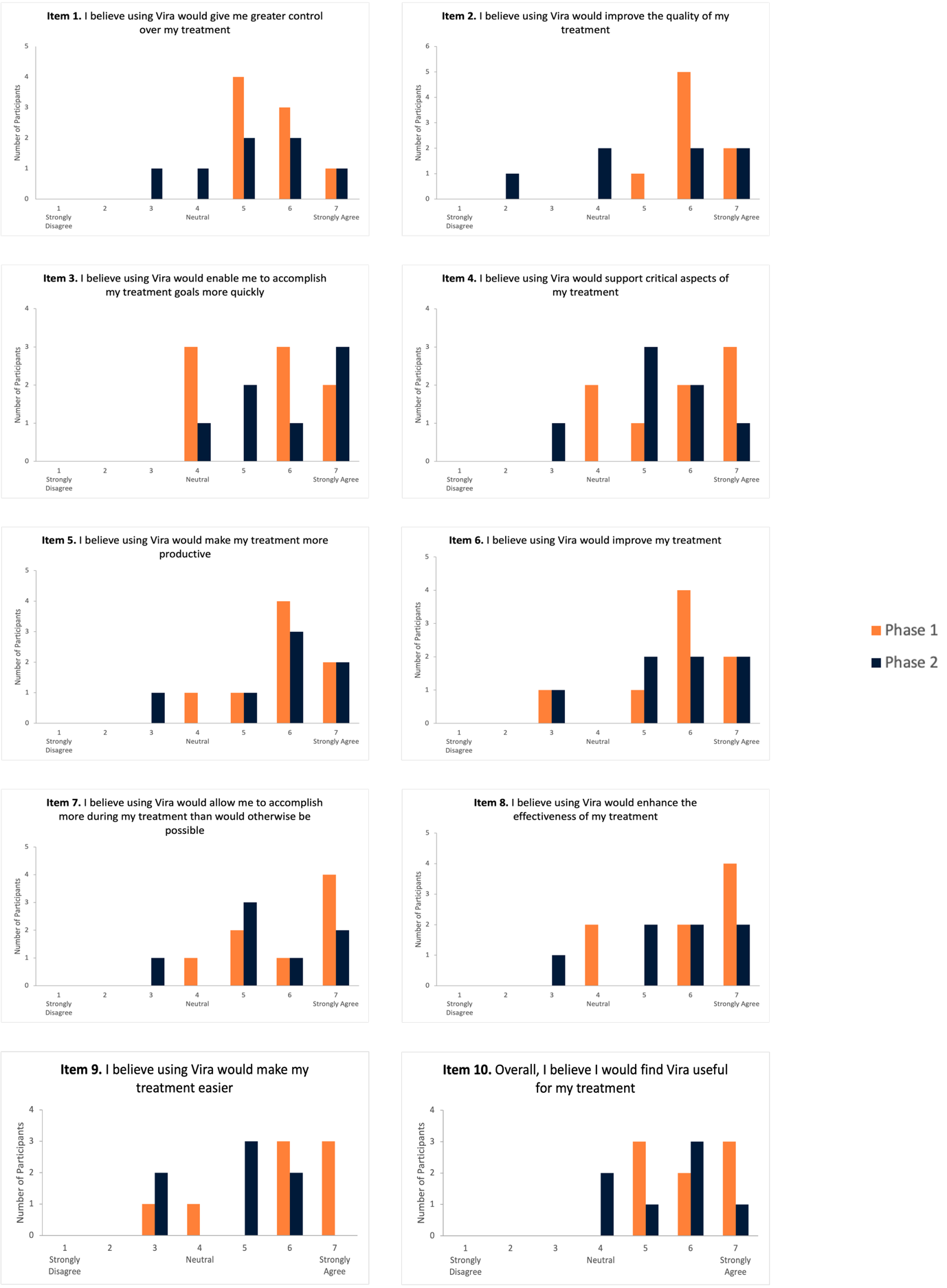


**Figure S2.** Distribution of adolescent ratings of perceived ease of use across phases.


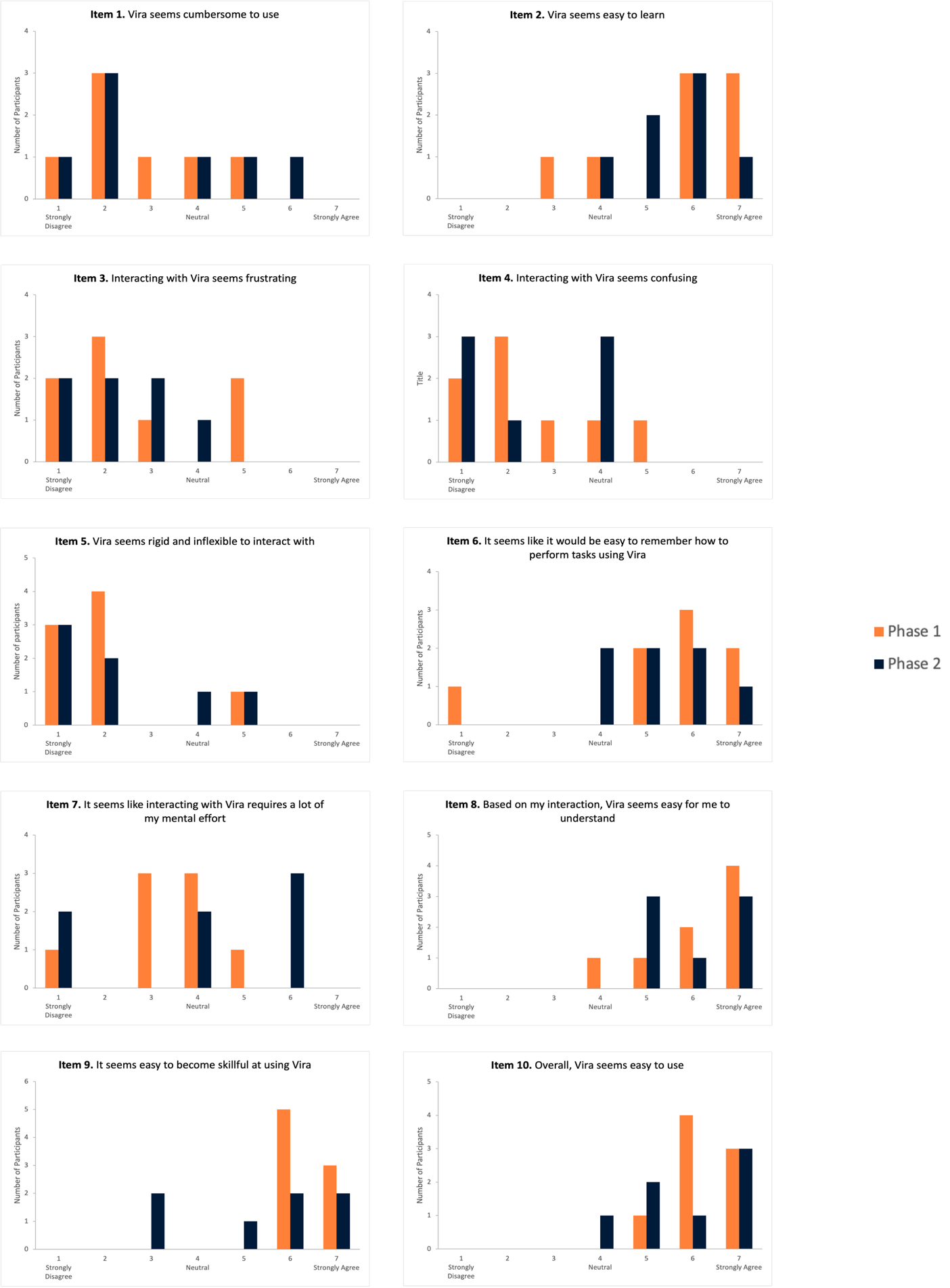


**Figure S3.** Distribution of clinician ratings of perceived usefulness across study phases.


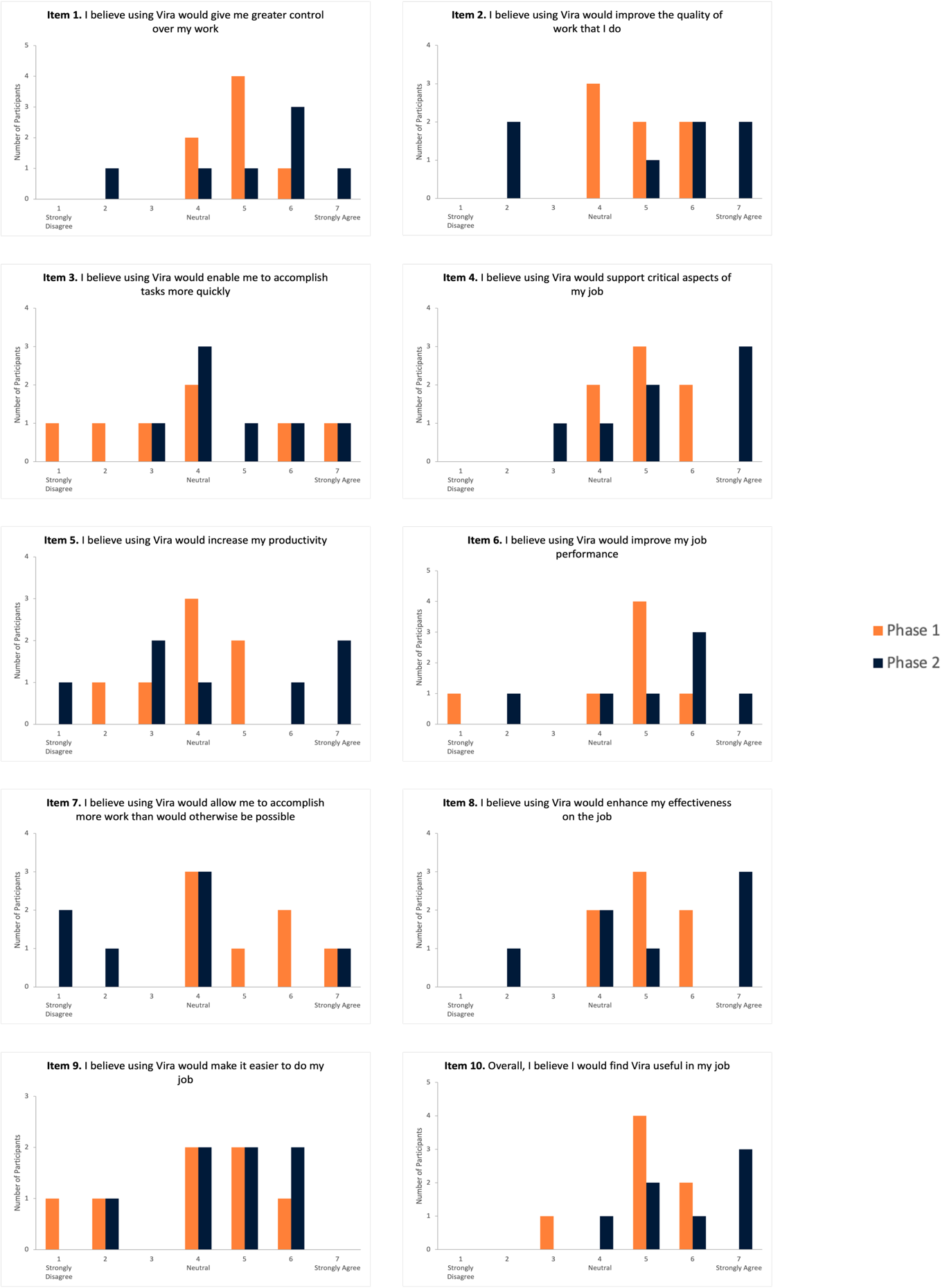


**Figure S4.** Distribution of clinician ratings of perceived ease of use across study phases.


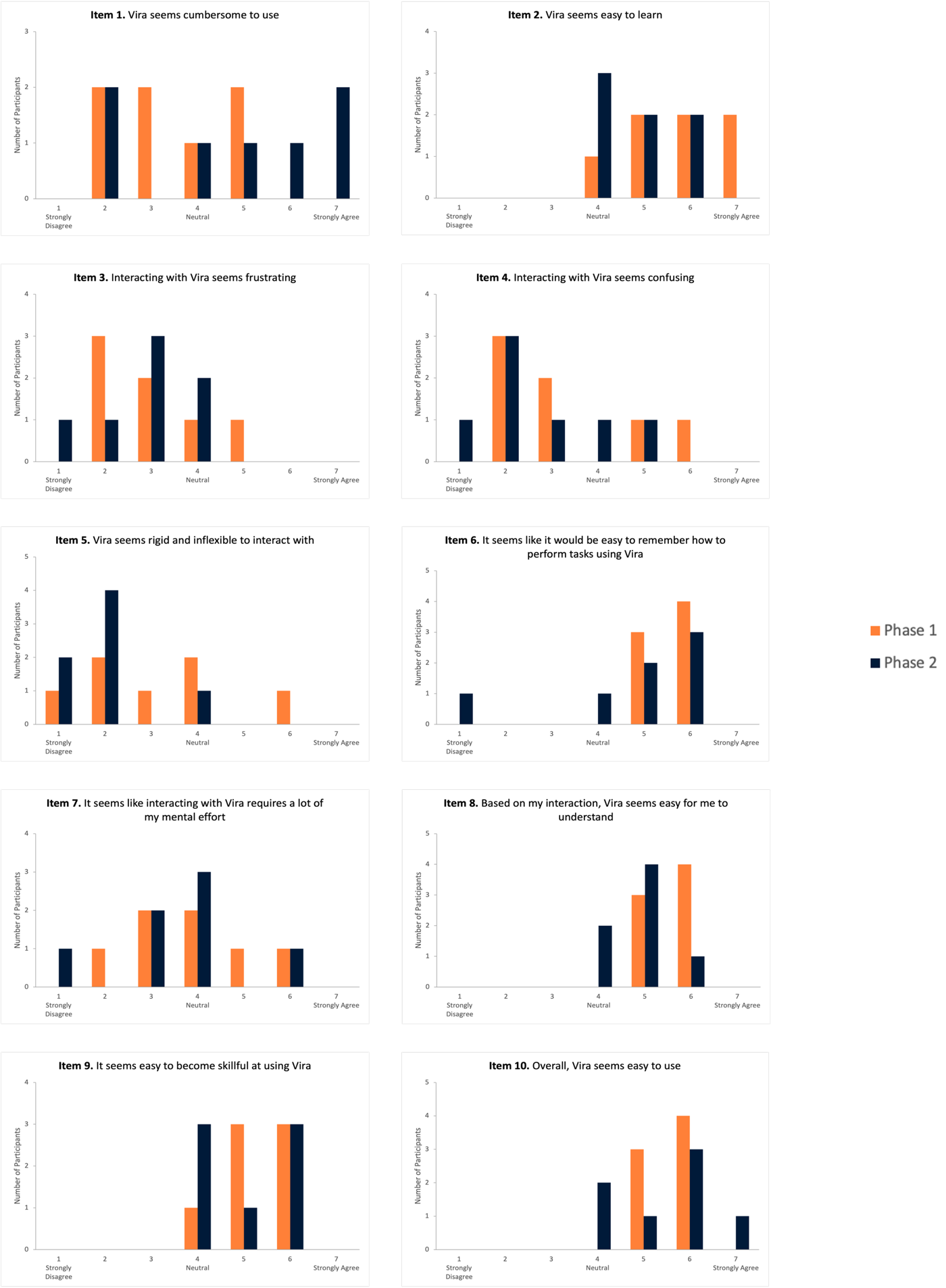


**Figure S5.** Distribution of business gatekeeper ratings of perceived usefulness.


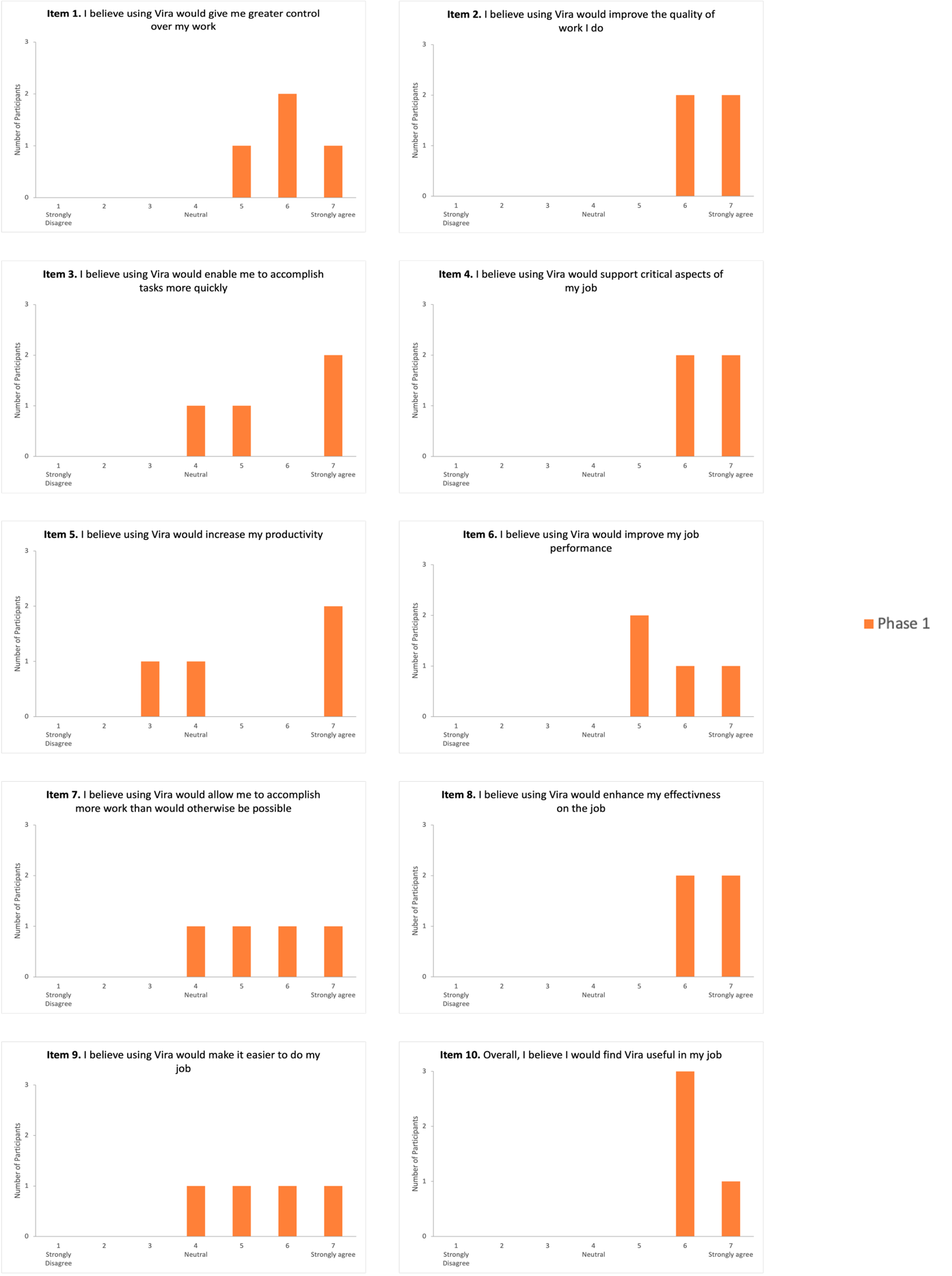


**Figure S6.** Distribution of business gatekeeper ratings of perceived ease of use.


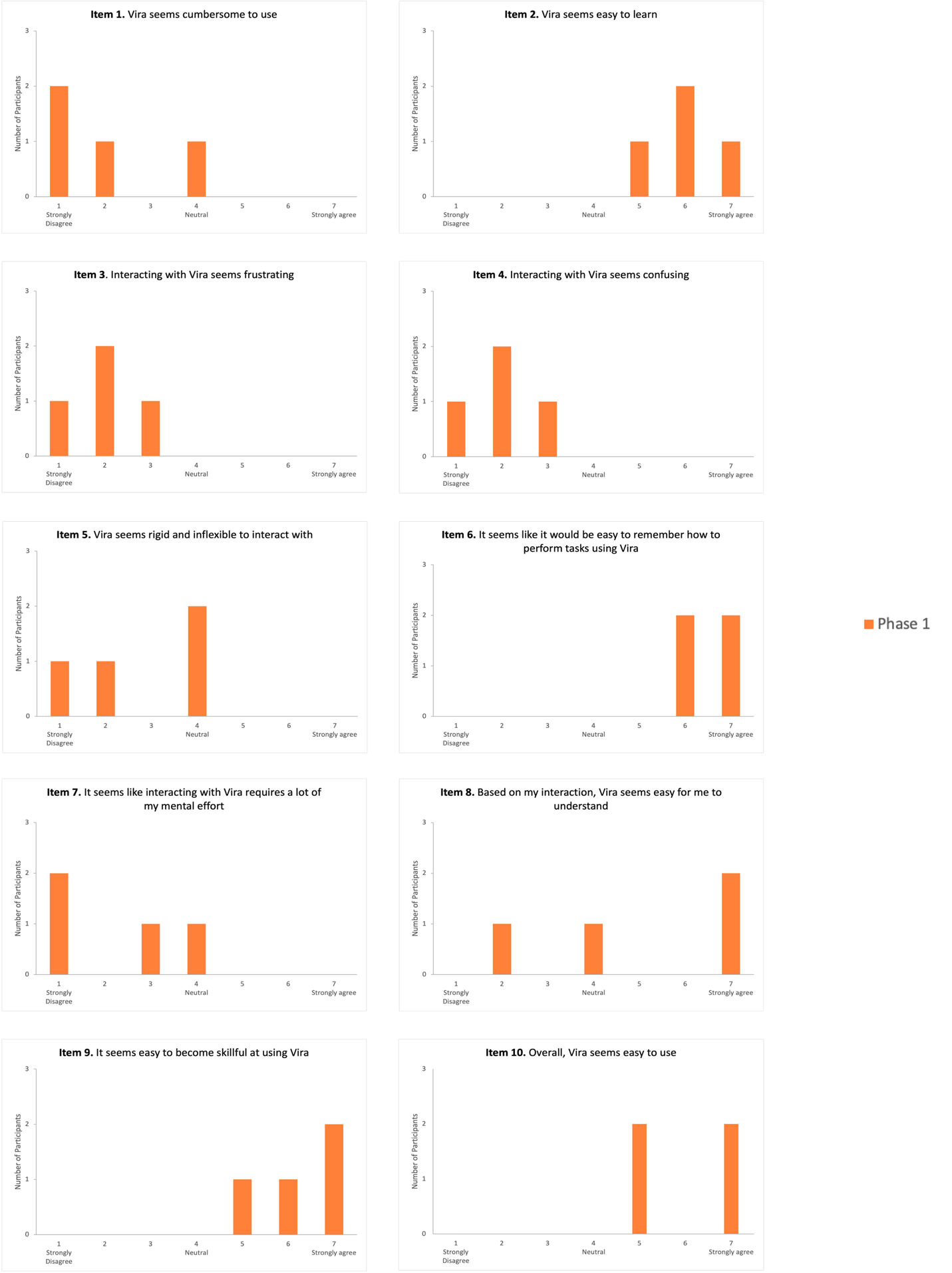

Supplement: Multimedia Appendix 1 [file formative_v9i1e65418_app1.docx]
